# Lateral Ventricle Solitary Fibrous Tumor: A Case Report and Review of the Literature

**DOI:** 10.7759/cureus.23106

**Published:** 2022-03-12

**Authors:** Anthony Nguyen, Yuan Shan, Kristopher Lyon, Awais Z Vance

**Affiliations:** 1 Neurosurgery, Baylor Scott & White Health, Temple, USA; 2 Pathology, Baylor Scott & White Health, Temple, USA

**Keywords:** craniotomy, neurosurgery, hemangiopericytoma, solitary fibrous tumor, intraventricular

## Abstract

Solitary fibrous tumors (SFTs) are rare tumors thought to be of mesenchymal origin. Even though intracranial, especially intraventricular, SFTs are rare, this diagnosis should be considered in the differential for intraventricular lesions. Here, report the case of a female in her 60s who underwent a non-contrast-enhanced magnetic resonance imaging scan of the brain for new-onset memory issues and headache which revealed a well-circumscribed intraventricular lesion in the right lateral ventricle with vasogenic edema, trapping of the temporal horn, and subfalcine herniation. She was admitted and started on dexamethasone prior to surgical treatment of the tumor. A right-sided superior parietal lobule approach was utilized to reach and resect the lesion. Histopathology was consistent with World Health Organization grade I SFT. Only 10 other cases of lateral ventricular SFTs have been reported in the literature. Intraventricular SFT is a rare diagnosis, and, as such, the literature on this topic mostly consists of case reports. Although the lesion is benign, metastases have been reported, and thus, gross total resection remains the standard of care. This case adds to the paucity of SFTs reported in the literature.

## Introduction

Solitary fibrous tumor (SFT) was first pathologically characterized in 1931 by Klemperer and Rabin [1]. Since then, advances in molecular biology and sequencing have allowed clinicians and researchers to discover that SFT encompasses tumors previously known as hemangiopericytomas (HPCs) [2]. As such, the term HPC has been retired by the World Health Organization (WHO), but the term “HPC phenotype” is often used to describe WHO grade II and III SFTs. Additionally, SFT/HPC is a term often used in the literature given the extensive use of the term HPC in the past.

Intracranial SFT is an uncommon diagnosis, and these tumors rarely originate in the cerebral ventricles [3]. SFT is of mesenchymal origin, and common differential considerations include meningioma, schwannoma, fibrosarcoma, and, when intraventricular, choroid plexus papilloma [4]. Historically, HPC was also considered a differential diagnosis but is now treated as a more malignant phenotype of SFT given their identical genetic signatures. Given the possibility of malignant transformation, clinician awareness of this entity is important [5].

Approximately half of intraventricular solitary fibrous tumors (ISFTs) originate in the lateral ventricles. Here, we report a rare case of an ISFT and review the literature for other cases of SFTs arising from the lateral ventricles.

## Case presentation

**Figure 1:**
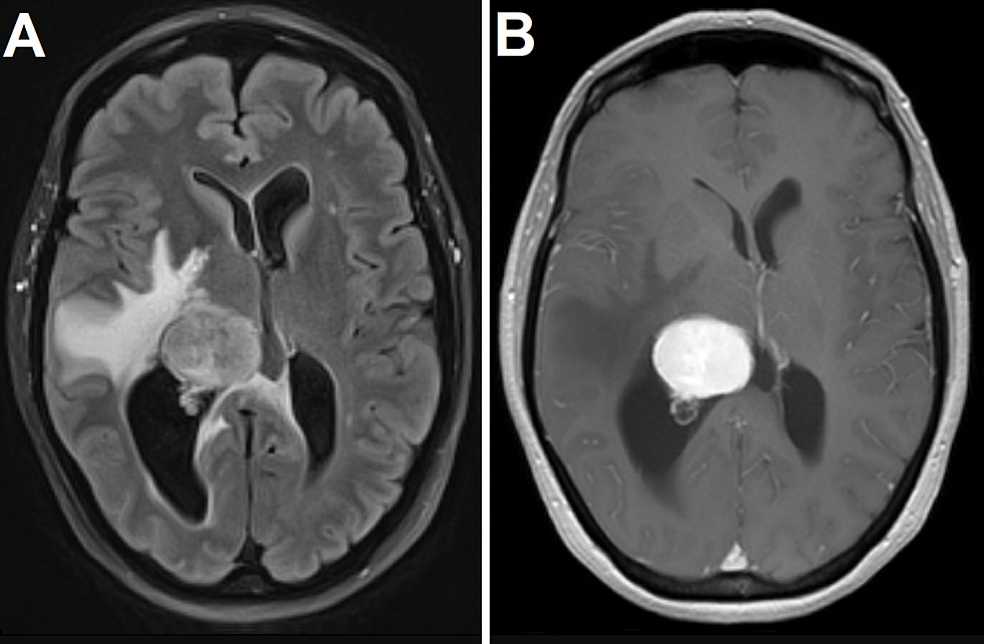
Magnetic resonance imaging of the brain with fluid-attenuated inversion recovery (A) and gadolinium-enhanced T1-weighted (B) images demonstrating an avidly contrast-enhancing, well-demarcated intraventricular tumor with associated vasogenic edema.

A 64-year-old female presented to the internal medicine clinic for two weeks of lightheadedness upon standing and memory issues regarding work she had performed for decades. She had also recently began developing headaches. Given her new-onset headaches and memory issues, a non-contrast-enhanced magnetic resonance imaging (MRI) scan of the brain was ordered. Following completion of the MRI, the patient was referred to the emergency department due to a finding of an intraventricular mass in the atrium of the right lateral ventricle with associated trapping of the temporal horn and subfalcine herniation (Figure 1A). The patient was admitted and started on dexamethasone. She was monitored closely neurologically. A contrast-enhanced MRI demonstrated homogenous contrast enhancement (Figure 1B). There was no evidence of metastatic disease in her chest, abdomen, or pelvis, and thus, metastasis was felt to be lower on the list of differential diagnoses. The most likely diagnoses were felt to be intraventricular meningioma and choroid plexus papilloma, although the patient’s age rendered the latter diagnosis less likely.

**Figure 2:**
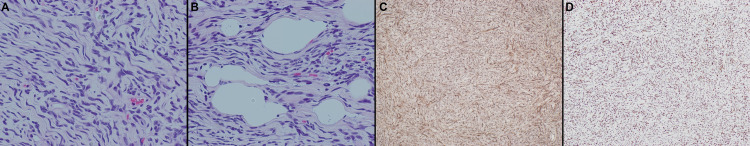
Hematoxylin and eosin stains under 200× magnification demonstrating spindle cells (A) and small, thin-walled vessels (B), which are characteristic of SFT. SFT is immunoreactive for CD34 (C). There is also strong nuclear expression of STAT-6 (D). SFT: solitary fibrous tumor; STAT-6: signal transducer and activator of transcription 6

**Figure 3:**
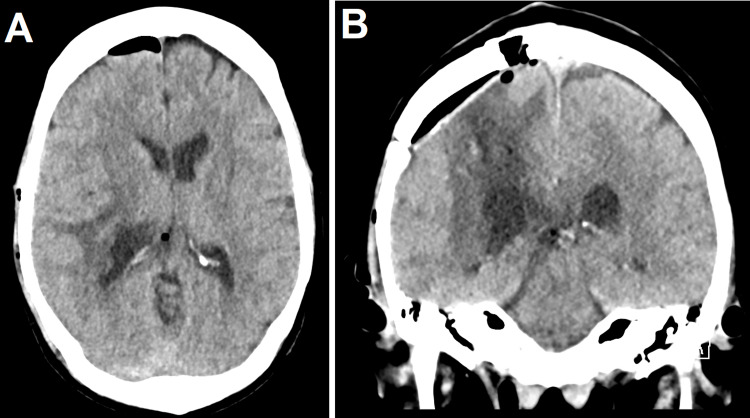
Postoperative axial (A) and coronal (B) slices of computed tomography scan of the head demonstrating expected post-surgical changes.

The option of surgical resection via a superior parietal lobule (SPL) approach was considered due to its ability to spare the sensorimotor cortex and optic radiations. This approach was discussed with the patient, and she provided informed consent to proceed. Stereotactic navigation was utilized to help with localization. A parietal craniotomy followed by an SPL corticotomy were performed in a standard fashion. The tumor was resected with the assistance of ultrasonic aspiration. The tumor was adherent at its base to the ventricle, which was also where its blood supply entered. The blood supply was coagulated and cut, and it was felt that gross total resection (GTR) was achieved. Final pathology was consistent with an ISFT, WHO grade I (Figure 2). A postoperative computed tomography scan of the head demonstrated expected post-surgical changes without complications (Figure 3). Given the low grade of the tumor and GTR, the decision was made to forego adjuvant radiotherapy, and a three-month follow-up MRI is planned in concordance with departmental protocol.

## Discussion

We describe a rare case of SFT arising in the lateral ventricles. Initial differential diagnoses included intraventricular meningioma (which was felt most likely), choroid plexus papilloma, ependymoma, glioma, and metastasis. The patient underwent an SPL approach for GTR of the lesion with pathologic confirmation of a diagnosis of SFT. She did not undergo adjuvant therapies.

A review of the literature was performed by querying the PubMed database utilizing the following search term: (“intraventricular” OR “ventric*”) and (“solitary fibrous tum*”). Of the 46 papers returned, nine studies [6-14] reported new cases of patients diagnosed with specifically SFTs (not HPC) originating in the lateral ventricles for a total of 10 cases (Table 1). ISFT was first described in 2003. The study by Tihan et al. included two cases of ISFT but did not specify the age or gender of the patients [14]. Of the remaining eight patients, half were female, and the average age was 57.5 years [7-13]. There was mention of choroid plexus attachment in four cases [7,8,11,13]. ISFTs arose in the ventricular trigone, and there was extension to the foramen of Monro in one case and to the third ventricle in one case. Another case report of ISFT stated that the lesion arose from the superior border of the cerebellum and exerted mass effect upon the lateral ventricle but does not specify which ventricle it originated from, so this report was excluded from Table 1 [15]. However, this case is notable in that there were multiple metastases to the acetabulum, iliac crest, thoracic vertebral body, and suprarenal perinephric fat. This patient underwent dacarbazine therapy with clinical improvement.

**Table 1:** Cases of lateral ventricle intraventricular solitary fibrous tumors reported in the literature, patient presentation, and subsequent treatment.

Study	Patient age/Gender	Presenting signs and symptoms	Treatment
Li et al. (2018) [6]	54 M	Not specified	Surgical resection, approach not specified
Bell et al. (2012) [7]	69 M	Gait disturbance, leg weakness, short-term memory deficit	Surgical resection, approach not specified
Vassal et al. (2011) [8]	60 F	Headache, Wernicke aphasia	Occipitoparietal craniotomy
Mekni et al. (2009) [9]	40 M	Not specified	Surgical resection, approach not specified
Boada et al. (2009) [10]	63 M	Facial paralysis	Surgical resection, approach not specified
Kinfe et al. (2008) [11]	75 F	Headache, gait disturbance, urinary incontinence, and short-term memory and attention deficits	Endoscopic frontal transcortical approach
Clarençon et al. (2007) [12]	44 F	Seizure, retro-orbital pain	Not specified
Surendrababu et al. (2006) [13]	55 F	Headache, vomiting, seizure	Middle temporal gyrus approach
Tihan et al. (2003) [14]	Not specified – two patients	Not specified	Surgical resection, approach not specified

Three surgical approaches were specified, and each differed from the other [8,11,13]. Vassal et al. reported the utility of an occipitoparietal craniotomy for an ISFT located in the atrium of the lateral ventricle [8]. Whether that patient underwent an interhemispheric approach or corticotomy is unspecified. Kinfe et al. reported the use of an endoscopic frontal transcortical approach for an ISFT located at the foramen of Monro, an approach commonly utilized for endoscopic third ventriculostomy [11]. Surendrababu et al. reported a middle temporal gyrus (MTG) corticotomy to access the lesion, which was adherent to the choroid plexus in the atrium of the lateral ventricle [13]. The cases reported by Vassal et al. and Surendrababu et al. most closely resembled our patient [8,13]. While the SPL, occipitoparietal interhemispheric, and MTG approaches can all provide access to the atrium of the lateral ventricle, the SPL and MTG approaches are more commonly employed. Of these two, the SPL approach is associated with a lower risk of postoperative visual deficits and permanent complications [16]. As such, the SPL approach is a safe and feasible option for resection of ISFT located in the lateral ventricular atrium.

ISFT is a rare diagnosis, and thus, management is based upon the few case reports and series available in the literature. Given the potential for malignant transformation and metastasis, surgical resection is the mainstay of treatment. Adjuvant radiation can be considered for more malignant phenotypes or for residual disease, and chemotherapy can be considered for metastatic disease. Physicians should be aware of this clinical entity given the possibility of progression.

## Conclusions

SFTs are uncommon intracranial lesions that can radiographically imitate more common tumors such as meningiomas, schwannomas, and choroid plexus papillomas. They are generally benign but can transform and exhibit malignant behaviors such as local invasion, significant locoregional mass effect, recurrence following GTR, and metastasis to distant locations. Although ISFT is a rare diagnosis, this diagnosis should be considered on the differential for intraventricular tumors (especially in an adult patient), and GTR should be attempted, when possible, to minimize the risk of future complications related to the tumor. The optimal surgical approach depends on the exact location of the tumor, but for SFTs located in the atrium of the lateral ventricle, the SPL approach is a safe and feasible surgical option, as are the occipitoparietal interhemispheric and middle temporal gyrus approaches.
